# Molecular and morphological identification of suspected *Plasmodium vivax* vectors in Central and Eastern Sudan

**DOI:** 10.1186/s12936-021-03671-9

**Published:** 2021-03-04

**Authors:** Omnia Fathelrhman Abdelwhab, Arwa Elaagip, Musab M. Albsheer, Ayman Ahmed, Giacomo Maria Paganotti, Muzamil Mahdi Abdel Hamid

**Affiliations:** 1 Department of Epidemiology, Tropical Medicine Research Institute, National Center for Research, Khartoum, Sudan; 2 Department of Parasitology and Medical Entomology, Faculty of Medical Laboratory Sciences, University of Khartoum, Khartoum, Sudan; 3 Department of Parasitology and Medical Entomology, Institute of Endemic Diseases, University of Khartoum, Khartoum, Sudan; 4 Botswana-University of Pennsylvania Partnership, Gaborone, Botswana; 5 Division of Infectious Diseases, Perelman School of Medicine, University of Pennsylvania, Philadelphia, PA USA; 6 Department of Biomedical Sciences, University of Botswana, Gaborone, Botswana

**Keywords:** Malaria vectors, *Anopheles funestus*, *Anopheles arabiensis*, *Anopheles pharoensis*, *Plasmodium vivax*, Circumsporozoite protein, Sudan

## Abstract

**Background:**

In spite of the global effort to eliminate malaria, it remains the most significant vector-borne disease of humans. *Plasmodium falciparum* is the dominant malaria parasite in sub-Saharan Africa. However, *Plasmodium vivax* is becoming widely spread throughout Africa. The overuse of vector control methods has resulted in a remarkable change in the behaviour of mosquito that feeds on human as well as on vector composition. The aim of this study was to identify *Anopheles* mosquito species in vivax malaria endemic regions and to investigate their role in *P. vivax* circumsporozoite protein (*Pvcsp*) allele diversity.

**Methods:**

Mosquito samples were collected from Central Sudan (Rural Khartoum and Sennar) and Eastern Sudan (New Halfa, Kassala state) using pyrethrum spray catch (PSC) and CDC light traps. Mosquitoes were identified using appropriate morphological identification keys and *Anopheles gambiae* complex were confirmed to species level using molecular analysis. A subset of blood-fed anopheline mosquitoes were dissected to determine the presence of natural infection of malaria parasites. In addition, the rest of the samples were investigated for the presence of *Pvcsp* gene using nested-PCR.

**Results:**

A total of 1037 adult anopheline mosquitoes were collected from New Halfa (N = 467), Rural Khartoum (N = 132), and Sennar (N = 438). Morphological and molecular identification of the collected mosquitoes revealed the presence of *Anopheles arabiensis* (94.2%), *Anopheles funestus* (0.5%), and *Anopheles pharoensis* (5.4%). None of the dissected mosquitoes (N = 108) showed to be infected with malaria parasite. Overall *P. vivax* infectivity rate was 6.1% (63/1037) by *Pvcsp* nested PCR. Co-dominance of *An. arabiensis* and *An. pharoensis* is reported in Sennar state both being infected with *P. vivax*.

**Conclusion:**

This study reported *P. vivax* infection among wild-caught anopheline mosquitoes in Central and Eastern Sudan. While *An. arabiensis* is the most abundant vector observed in all study areas, *An. funestus* was recorded for the first time in New Halfa, Eastern Sudan. The documented *Anopheles* species are implicated in *Pvcsp* allele diversity. Large-scale surveys are needed to identify the incriminated vectors of *P. vivax* malaria and determine their contribution in disease transmission dynamics.

## Background

The estimated malaria cases in 2019 was 229 million cases occurred worldwide resulting in 409,000 malaria related death, owing the deadliest parasite (*Plasmodium falciparum*), predominantly in sub-Saharan Africa [1]. According to the World malaria report in 2020, there were 2015 million cases in 2019, mostly (94% of total cases) in African continent [1]. *Plasmodium falciparum* is considered to be the most important malaria species responsible for more than 99.7% of malaria cases [2] followed by *P. vivax* [3], a generally considered less pathogenic parasite causing a benign type of malaria. However, the “benign tertian malaria” description of vivax malaria has been challenged by recent reports and documentation of severe *P. vivax* infections and even deaths [4, 5]. *Plasmodium vivax* stands for about half infections outside Africa [6–8], representing 75% of malaria cases in the WHO Region of the Americas, 53% in the WHO Region of South-East Asia [1], and 40% in the Eastern Mediterranean Region [7]. However, its presence in Africa has not well documented and reported because of the very high endemicity of *P. falciparum* and for the accepted paradigm that Africans are protected from *P. vivax* infection by genetic factors [9, 10].

*Plasmodium vivax* parasite exploits the human Duffy antigen/chemokine receptor (DARC) to invade the red blood cell [11]. Duffy antigen is rarely expressed in the African populations [12], so infection prevalence was thought to be less in Africa due to negativity of Duffy binding protein among its population [8, 13]. However, several studies revealed that infection may persist in individuals lacking this receptor [14, 15].

In Sudan, *P. falciparum* is responsible for 91.2% of malaria cases while *P. vivax* makes 8.8% of the cases [16, 17]. However, during the recent years, the number *P. vivax* infections is increased throughout the country with an overall prevalence of 26.6% [18], and prevalence of 40%, 38% in White Nile and Gezira states, respectively [15, 19]. The role of *Anopheles* mosquitoes in transmitting malaria parasites depends on several factors including their preference to feed on humans [20] and their innate susceptibility to the *Plasmodium* [21, 22]. The main malaria vectors in Africa belong to three major groups of vectors, the *Anopheles gambiae* complex, the *Anopheles funestus* group, and the *Anopheles nili* complex [23, 24]. Methods of mosquito control still rely heavily on the use of long-lasting insecticidal nets (LLINs) and indoor residual spraying (IRS) [25, 26], that target indoor resting vectors. An updated study of anopheline mosquitoes and their behaviour is much needed to guide the vector control operations [27, 28].

The vast majority of studies stated that *Anopheles arabiensis* is the main if not the only malaria vector in Sudan [29–32]. However, few studies encountered the presence of other *Anopheles* species at malaria foci in Sudan, such as *An. funestus *sensu stricto (*s.s.*) [33], *Anopheles pharoensis* [34] and *An. nili *sensu lato (*s.l*.) [35].

*Plasmodium* circumsporozoite protein (CSP) is an abundant surface protein expressed on the surface of sporozoite and oocyst [36] its expression starting on day seven onwards post mosquito infection [37]. This protein is implicated in salivary gland invasion in mosquitoes, sporozoite maturation, and hepatocyte invasion in humans [38]. *Plasmodium vivax* circumsporozoite (*Pvcsp*) gene has three distinctive variants (VK 210, VK 247, and *P. vivax-*like) [39–41].

The present study was conducted to identify the anopheline mosquito fauna in regions where *P. vivax* malaria is endemic and to investigate their possible role in *P. vivax* circumsporozoite protein (*Pvcsp*) gene allele diversity.

## Methods

### Study areas and mosquito collection

**Fig. 1:**
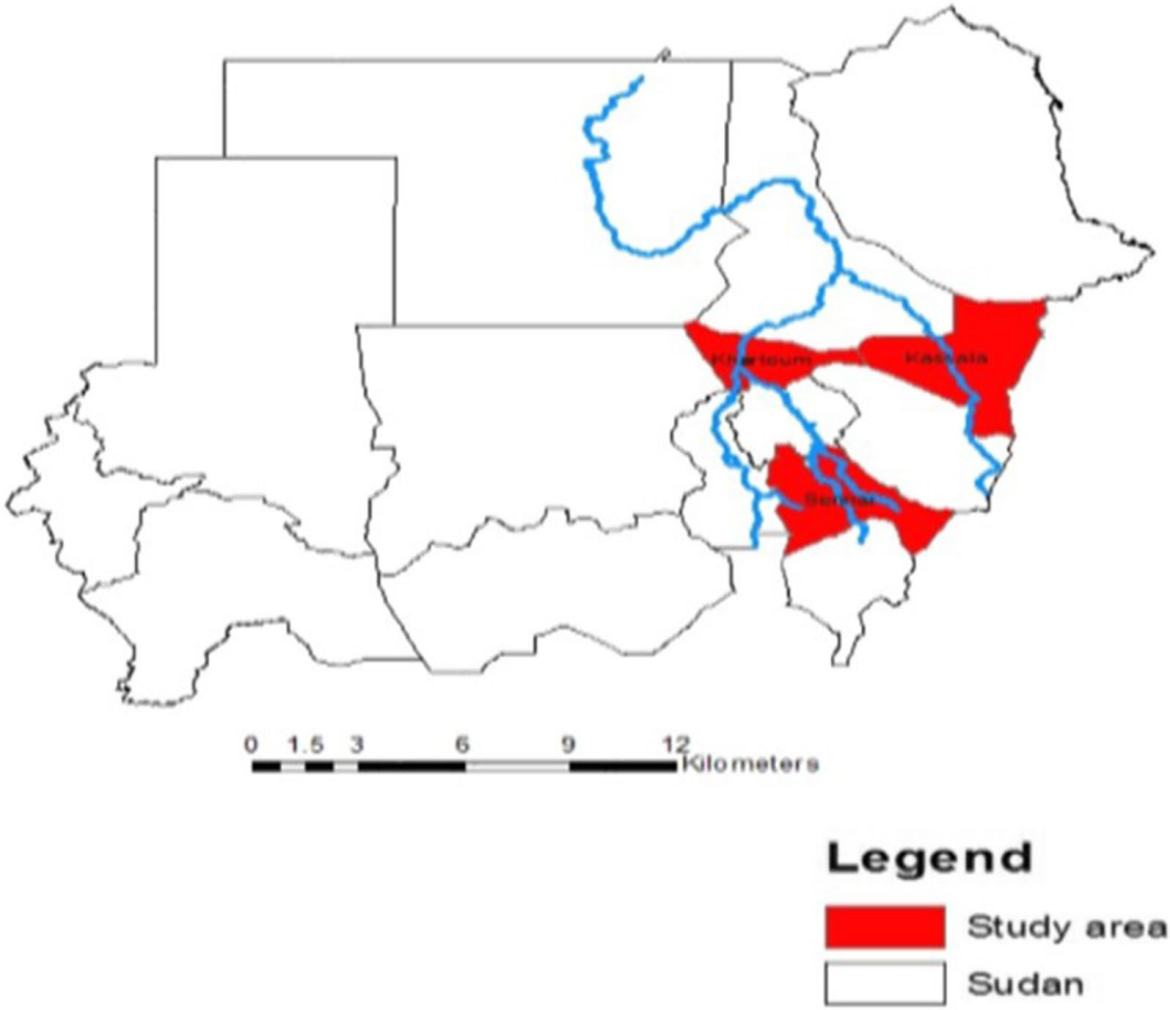
Local map of the study sites in Sudan. The areas highlighted in red represent the study areas of Rural Khartoum and Sennar (Central Sudan) and New Halfa (Eastern Sudan)

The study was conducted in two regions, Central Sudan (Gezura Slanj, Rural Khartoum state 15.7938° N, 32.8987° E, Sennar state 13.0317° N, 33.9750°) and Eastern Sudan (New Halfa, Kassala state 15.3288° N, 35.5986° E) (Fig. 1). The collection of the adult anopheline mosquito samples was carried out between September 2015 to February of 2016 using standard Pyrethrum Spray Catch (PSC), and CDC light traps. In each study area, two endemic malaria sites were chosen randomly for mosquito adult collection. The collection period was three consecutive nights per month per site.

### Samples processing and morphological identification

The collected adult mosquitoes were sorted out according to genus level. All female anopheline mosquitoes were divided into blood-fed, unfed, and gravid. A subset of blood-fed samples, collected by CDC light traps, was kept alive and dissected immediately using dissecting stereomicroscope and sterilized dissecting needles to detect the natural infection of *Plasmodium* parasites according to [42], while the rest of the samples, in addition to carcasses of freshly dissected samples, were preserved as dry specimens in labelled eppendorf tubes containing silica gel and stored at room temperature until morphological identification and molecular analysis. Samples were identified to species level using standard entomological keys [23, 43].

### DNA extraction and *Pvcsp* nested-PCR

Genomic DNA was extracted from whole individual mosquito using the Livak method [44]. Samples of *An. gambiae* complex were subjected to molecular identification following the protocol of Scott et al*.* [45], with slight modification on the cycling number to be set as 36 cycles.

Nested-PCR was performed to detect *Pvcsp* gene following [46, 47] with minor modifications. Cycling condition for the outer PCR was as follows: 95 ºC initial denaturation for 3 min, 37 cycles of: denaturation at 94 ºC for 30 s, annealing at 58 ºC for 1 min, 72 ºC elongation for 1 min, and final elongation was performed at 72 ºC for 10 min. The nested PCR was set as follows: 95 ºC initial denaturation for 3 min, 15 cycles of: 94 ºC for 30 s, 63.8 ºC for 1 min, 72 ºC for 2 min, cycles of: 94 ºC for 30 s, 64 ºC for 1 min, 72 ºC for 2 min and final elongation at 72 ºC for 10 min.

PCR products were separated in 1% agarose gel stained with ethidium bromide and observed under UV using BioDocAnalyze gel image documentation system (Biometra Analytika Jena Company, Germany).

## Results

A total of 1037 adult female anopheline mosquitoes were collected from three study sites; New Halfa (N = 467), Rural Khartoum (N = 132), and Sennar (N = 438). Out of the total, 644 mosquitoes were blood-fed, 393 were unfed, while 117 were gravid (Table 1). The combined morphological and molecular identification revealed that the species composition of the collected mosquito was predominately *An. arabiensis* 94.2% (976/1037), *An. pharoensis* 5.4% (56/1037), and *An. funestus s.s.* 0.5% (5/1037) (Table 1).

**Table 1:** Represents anopheline adult mosquito collected from different study areas in Central and Eastern states, Sudan during September 2015 – February 2016

Study area	Total anopheline collected	Physiological status			Species identification (N)	*P. vivax* infectivity rate n (%)
		Blood-fed	Unfed	Gravid		
New Halfa	467	214	201	52	*Anopheles arabiensis* (462)	57 (12.2%)
					*An. funestus* (5)	0 (0%)
Rural Khartoum	132	113	0	19	*An. arabiensis* (132)	0 (0%)
Sennar	438	298	94	46	*An. arabiensis* (382)	5 (1.1%)
					*An. pharoensis* (56)	1 (0.2%)
Total	1037	625	295	117	1037	63

A subset (N = 108) of blood-fed mosquitoes were dissected to detect natural infection with malaria parasite (*P. falciparum* and *P. vivax*). None was found infected with sporozoites. CSP gene analysis showed that sixty-three samples showed presence of *Pvcsp* gene, with overall infectivity rate (6.1%). Details of numbers of infected mosquito species among study sites are presented in Table 1.

**Fig. 2:**
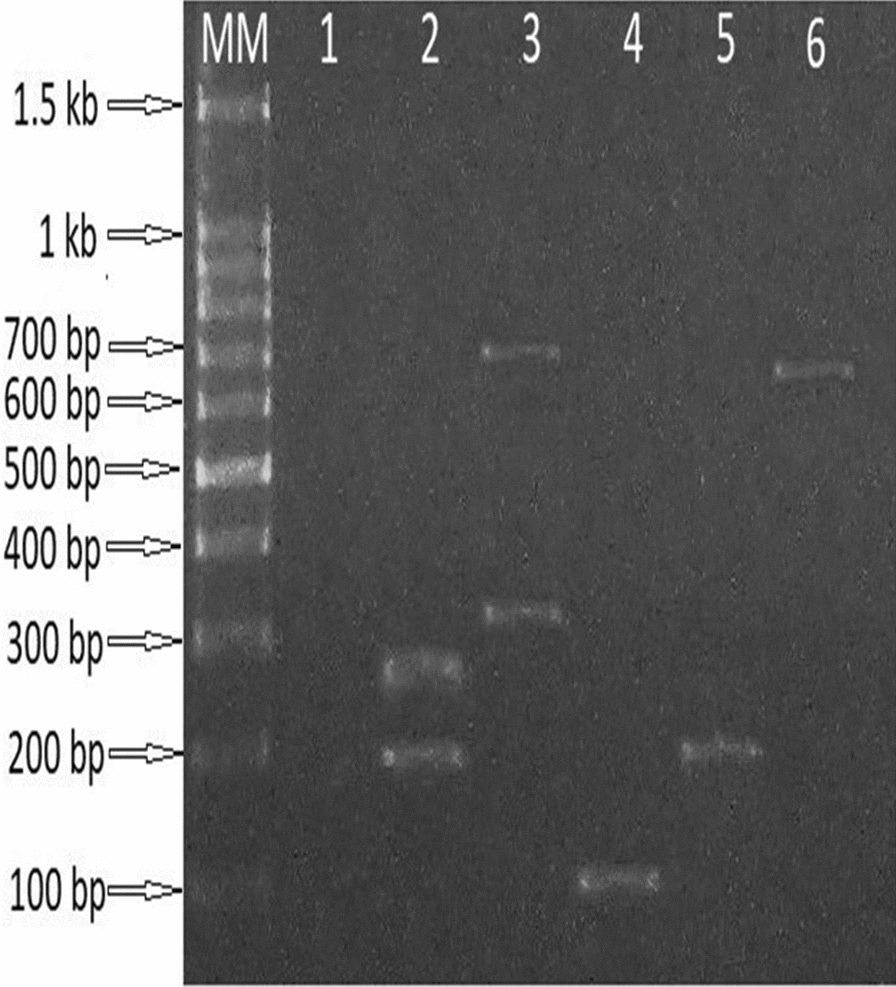
1.5% Agarose gel showing amplification of *Plasmodium vivax* circumsporozoite gene (*Pvcsp)* allelic variants using VCS1F/VCS1R outer and VCS2F/VCS2R nested primers. MM: 100 bp DNA ladder; lane 1: negative control; Lane 2–6: positive samples

Six *Pvcsp* allelic variants were found in two study areas. In New Halfa, five allelic variants (700 bp, 300 bp, 250 bp, 200 bp and 100 bp) were detected (Fig. 2), while in Sennar state, two allelic variants (400 bp and 300 bp) were detected in mosquito samples (Fig. 2).

## Discussion

The present study was conducted to identify the species of *Anopheles* in regions endemic for *P. vivax* malaria and to investigate their role in *P. vivax* circumsporozoite protein (*Pvcsp*) gene allele diversity.

Updating the existed knowledge about the vector composition and relative density in malaria endemic areas are essential entomological and epidemiological indicators for the disease burden, transmission season, and monitoring the vector control methods [48–50].

Previous studies in Sudan [5, 31, 51] reported that *P. falciparum* is responsible for more than 95% of clinical malaria cases while revealing 3% were due to *P. vivax.* However, recently there an increasing numbers *P. vivax* cases reported in many parts of the country [18].

Results of this study demonstrated that *An. arabiensis* was the most abundant *Anopheles* species, followed by *An. pharoensis,* and the least was *An. funestus*, supporting that *An. arabiensis* is the principal malaria vector in Sudan. For the first time, *An. pharoensis* was found positive for *P. vivax.* In addition to Lewis in 1956 [35], no further published data from Sudan had suggested a role for *An. pharoensis,* which is considered to be a secondary vector. Other studies in Sennar [31], and Gezira state [30] reported *An. arabiensis* to harbouring malaria parasites. In contrast to Himeidan et al. and Lewis [52, 53], who had recorded the presence of *An. pharoensis* and *Anopheles multicolour* in New Halfa, this study showed the presence of *An. funestus* in the same area but in a small number.

In this study, sporozoites were not observed in the dissected blood fed mosquitoes. This result is similar to previous studies conducted in Sennar and Khartoum [31, 54]. One explanation for this finding could be due to the fact that fresh blood fed mosquitoes may have been freshly infected by early stages of *Plasmodium* parasites during their sexual life cycle such as mature gametes, zygotes, ookinetes, oocysts. Appearance of sporozoites usually requires approximately two weeks from the time of ingesting infected blood meal with malaria parasites by mosquito vectors [37].

In this study, *P. vivax* was not detected in any mosquito collected in Rural Khartoum in accordance with a previous study conducted in Khartoum state [55]. In the present study, six distinct variants of *P. vivax* were identified (five alleles and two alleles in New Halfa Sennar, repectively) and a marked difference in infectivity rates of the identified mosquitoes (*An. arabiensis versus An. pharoensis*) was demonstrated in the two study sites. This variation in *Pvcsp* allelic distribution perhaps is due to adaptation of parasites to local mosquito species thus transmit distinct *P. vivax* variants/haplotypes with different efficiency [56, 57]. A better understanding of co-evolutionary dynamics between co-dominant mosquitoes and parasites will facilitate the identification of molecular mechanisms related to disease transmission and provide important data to guide malaria control. *Plasmodium vivax* is becoming a serious health problem exhibiting a wide range of hosts children adults and even pregnant women [58]. It has also been detected in asymptomatic individuals [59, 60]. The underestimation of *P. vivax* malaria infections could be attributed to misdiagnosis of the infection using rapid diagnostic tests (RDTs) [61], or the presence of hypnozoites which cannot be detected using RDTs [62].

Assessment of the impact of vector control interventions on malaria transmission requires more data about entomological indicators including the identification of the vector composition, distribution, and density [63].

## Conclusion

The findings of this study are very alarming mainly because it showed the expansion of the efficient malaria vector distribution, *An. funestus,* into Eastern Sudan. The study also confirms the role of *Anopheles* species in *Pvcsp* allele diversity in Sudan. These findings suggest changes in malaria epidemiology in Sudan that requires further entomological, parasitological, and epidemiological studies to accurately determine the distribution and density of malaria vectors countrywide, and to investigate their role in the malaria transmission. Additionally, the potential association between vector species and different *Plasmodium* species they transmit, need to be investigated thoroughly. Furthermore, the susceptibility of the malaria vectors in Sudan to the currently applied vector control tools must be urgently investigated.

## Data Availability

All data generated or analyzed during this study are included in this published article.

## Abbreviations

PSC: Pyrethrum Spray Catch
s. s.: Sensu stricto
s. l.: Sensu lato
CSP: Circumsporozoite protein
Pvcsp: *Plasmodium vivax* Circumsporozoite protein
WHO: World Health Organization
DARC: Duffy antigen/chemokine receptor
LLINs: Long-lasting insecticidal nets
IRS: Indoor residual spraying
SNP: Single nucleotide polymorphism
IGS: Intergenic spacer region
PCR: Polymerase chain reaction
UV: Ultra violet
RDTs: Rapid diagnostic tests
VK: *P. vivax* Csp gene variant
